# Assessment of Healthcare Workers’ Levels of Preparedness and Awareness Regarding COVID-19 Infection in Low-Resource Settings

**DOI:** 10.4269/ajtmh.20-0330

**Published:** 2020-06-18

**Authors:** Muhammed Elhadi, Ahmed Msherghi, Mohammed Alkeelani, Abdulaziz Zorgani, Ahmed Zaid, Ali Alsuyihili, Anis Buzreg, Hazim Ahmed, Ahmed Elhadi, Ala Khaled, Tariq Boughididah, Samer Khel, Mohammed Abdelkabir, Rawanda Gaffaz, Sumayyah Bahroun, Ayiman Alhashimi, Marwa Biala, Siraj Abulmida, Abdelmunam Elharb, Mohamed Abukhashem, Moutaz Elgzairi, Esra Alghanai, Taha Khaled, Esra Boushi, Najah Ben Saleim, Hamad Mughrabi, Nafati Alnafati, Moaz Alwarfalli, Amna Elmabrouk, Sarah Alhaddad, Farah Madi, Malack Madi, Fatima Elkhfeefi, Mohamed Ismaeil, Belal Faraag, Majdi Badi, Ayman AL-Agile, Mohamed Eisay, Jalal Ahmid, Ola Elmabrouk, Fatimah Bin Alshiteewi, Hind Alameen, Hala Bikhayr, Tahani Aleiyan, Bushray Almiqlash, Malak Subhi, Mawada Fadel, Hana Yahya, Safeya Alkot, Abdulmueti Alhadi, Abraar Abdullah, Abdulrahman Atewa, Ala Amshai

**Affiliations:** 1 Faculty of Medicine, University of Tripoli, Tripoli, Libya;; 2 Faculty of Medicine, University of Benghazi, Benghazi, Libya;; 3 Faculty of Medicine, Sebha University, Sebha, Libya;; 4 Faculty of Medicine, University of Zawia, Az-Zawiyah, Libya;; 5 Faculty of Medicine, University of AL-Mergib, Al Khums, Libya;; 6 Faculty of Medicine, Al-Zintan University, Az-Zintan, Libya;; 7 Faculty of Medicine, Misurata University, Misurata, Libya;; 8 Faculty of Medicine, Al-Jabal Al Gharbi University, Gherian, Libya

## Abstract

COVID-19, caused by the SARS-CoV-2 virus, is spreading rapidly worldwide, with devastating consequences for patients, healthcare workers, health systems, and economies. As it reaches low- and middle-income countries, the pandemic puts healthcare workers at high risk and challenges the abilities of healthcare systems to respond to the crisis. This study measured levels of knowledge and preparedness regarding COVID-19 among physicians and nurses. A cross-sectional survey was conducted among healthcare workers in Libya between February 26 and March 10, 2020. We obtained 1,572 valid responses of a possible 2,000 (78.6%) participants from 21 hospitals, of which 65.1% were from physicians and 34.9% from nurses. The majority of participants (70%) used social media as a source of information. A total of 47.3% of doctors and 54.7% of nurses received adequate training on how to effectively use personal protective equipment. Low confidence in managing suspected COVID-19 patients was reported by 83.8% of participants. Furthermore, 43.2% of healthcare workers were aware of proper hand hygiene techniques. Less than 7% of participants received training on how to manage COVID-19 cases, whereas 20.6% of doctors and 26.3% of nurses felt that they were personally prepared for the outbreak. Awareness and preparedness for the pandemic were low among frontline workers during the study. Therefore, an effective educational training program should be implemented to ensure maintenance of appropriate practices during the COVID-19 pandemic.

## INTRODUCTION

In late 2019, a novel COVID-19 was reported to cause severe viral pneumonia in Wuhan, China. It has since spread worldwide, resulting in a pandemic that has now infected more than 1.3 million people, causing more than 80,000 deaths globally.^1,2^ In February 2020, the WHO named the condition COVID-19, which stands for coronavirus disease 2019.^3^

The severity of symptomatic infections ranges from mild to critical. Approximately 80% of patients have mild symptoms, whereas less than 20% experience severe symptoms such as dyspnea and shock; respiratory failure occurs in less than 5% of patients.^4–7^ Elderly patients and/or patients with comorbidities, such as cardiovascular diseases, respiratory diseases, hypertension, diabetes mellitus, and chronic kidney diseases, are at a higher risk for severe illness. They have a higher risk of mortality than younger, or otherwise healthier, individuals.^8,9^ Previous reports have confirmed that hospitalized patients had a mean age of 49–55 years.^6,10^ In an earlier report provided by the United States, regarding COVID-19 patients treated between February 12, 2020 and March 2020, about 67% of those hospitalized were older than 45 years, which is similar to a prior report from China.^11,12^

Acute respiratory distress syndrome (ARDS) is a significant complication for COVID-19 patients; an estimated 20–41% of patients develop ARDS following a COVID-19 infection and require mechanical ventilation.^5,13^ This characteristic of the disease can significantly increase the existing burden on healthcare facilities, and it requires extra resources and appropriate management.

COVID-19 poses a higher risk for physicians and nurses who work in critical care, emergency medicine, infectious diseases, and pulmonary medicine departments. Personal protective equipment (PPE), proper handwashing, and hand hygiene are critical in decreasing the transmission and risk of infection of COVID-19 in hospitals. Therefore, adequate training, knowledge, and resources are necessary to prevent hospital-acquired infections due to cross-contamination to other patients who receive care in these departments.^14–16^

As of April 12, 2020, the Libya National Center for Disease Control (NCDC) reported 524 suspected cases of COVID-19, with 25 confirmed diagnoses.^17^ However, these numbers raise a question regarding the local authorities’ ability to perform adequate testing for COVID-19, as the Libya NCDC can perform an average of 30 tests per day. Furthermore, given the current civil war, limited financial resources, and a shortage of machines and materials, Libya is more vulnerable to the COVID-19 pandemic. In 2017, the WHO released a report on the healthcare system in Libya, revealing that approximately 20% of hospitals were shut down. In addition, several major hospitals only operate at 80% capacity or less. The report states that chronic respiratory disease readiness is less than 29%, whereas the readiness of emergency services is less than 47% of the target. Furthermore, readiness for emergency health services is less than 18% in terms of training and 40% for diagnosis abilities. The report indicates that Libya does not have a rapid response team or the ability to diagnose and respond to alerts in an organized manner.^18^ This raises concerns about the local healthcare authorities’ preparedness and capacity to provide an adequate response to COVID-19 infection.

Therefore, critical preparedness, readiness, and knowledge regarding COVID-19 are needed for physicians and nurses on the front line.^16,19^ Few researchers have addressed the overall issues of preparedness of healthcare systems for COVID-19, especially in developing countries, where resources and facilities are limited. Considerable concerns have been raised regarding countries’ preparedness for COVID-19 and their ability to maintain control. This study aimed to determine the preparedness and knowledge of Libyan healthcare workers regarding COVID-19, and to develop and validate a measurement tool for estimating healthcare preparedness on a global scale.

## METHOD

### Study design.

A cross-sectional survey study was conducted in 21 hospitals in Libya between February 26, 2020 and March 10, 2020.

### Setting and participants.

The study was conducted among physicians and nurses working in the emergency department, intensive care units (ICUs), and respiratory and infectious disease departments, all of whom may expect to encounter COVID-19 patients. The study methodology was explained to the participants, and they were asked to provide consent before participating in the study. Doctors and nurses working in other departments or private clinics were excluded from the study. A total of 2,000 targeted participants were given a paper-based questionnaire at their workplace.

### Instruments.

The self-administered and anonymous questionnaire was developed by the authors of the study and was validated in a pilot study of 30 participants, who did not participate in the final analysis. Some of the questions were based on a framework similar to that of previous studies on infectious disease outbreaks.^20–22^ The structured questionnaire was validated to address questions about healthcare workers’ level of preparedness and knowledge of COVID-19 (Supplemental files I–II).

The first part of the survey was designed to collect data regarding the background characteristics of participants (age, gender, department, years of experience, information sources, and previous experience with outbreaks). The second part comprised seven items intended to evaluate the knowledge and awareness of physicians and nurses regarding COVID-19 infection. The third part comprised 11 items to assess the overall preparedness in terms of managing cases of COVID-19 infection. The questionnaire evaluates information sources, training experience with COVID-19, diagnosis and management of COVID-19 patients, use of PPE, safety precautions, isolation procedures, measures to prevent infection, and reporting procedures. To assess a given participant’s knowledge, each correct answer was given a score of 1, and an incorrect answer was given a zero. Scores were summed for a total score of 7, ranging from 0 to 7 for knowledge. The preparedness ranges from 0 to 11. Those who scored ≥ 5 on the knowledge scale were deemed to have a high level of knowledge, whereas those who scored ≥ 8 on the preparedness scale had adequate preparedness.

The questionnaire was developed in English for doctors and was translated into Arabic for nurses using a forward-backward translation by two qualified, independent linguistic translators at the University of Tripoli. Each translator independently performed a forward translation of the original questionnaire. Backward translation from Arabic to English was carried out by another linguistic translator. Three researchers then reviewed the translated versions of the questionnaire for discrepancies. Pilot tests of both versions were carried out, and Cronbach’s alpha was used to measure the internal consistency reliability of both versions. Participants were recruited in relative proportion to the estimated number of doctors and nurses working in these departments in Libya. We assumed that 70% of participants had basic knowledge and preparedness regarding COVID-19, and the estimated sample size was calculated with 80% power at a 95% confidence limit, with a design effect equal to 2, and using 20 clusters.

### Statistical analysis.

Data entry and analyses were performed using SPSS (IBM SPSS Statistics for Windows, version 25.0; IBM Corp., Armonk, NY). Descriptive statistics were used to describe the study variables. Frequency, percentage, and mean scores were used to report the descriptive analysis. The chi-square test was used to assess the association difference between the groups. Statistical significance was considered for *P* < 0.05.

### Ethical approval.

Ethical approval for this study was obtained from the Bioethics Committee at Biotechnology Research Center in Libya [Reference number: BEC-BTRC-109.3-2020]. All participants provided consent before participating in the study.

## RESULTS

**Figure 1.**
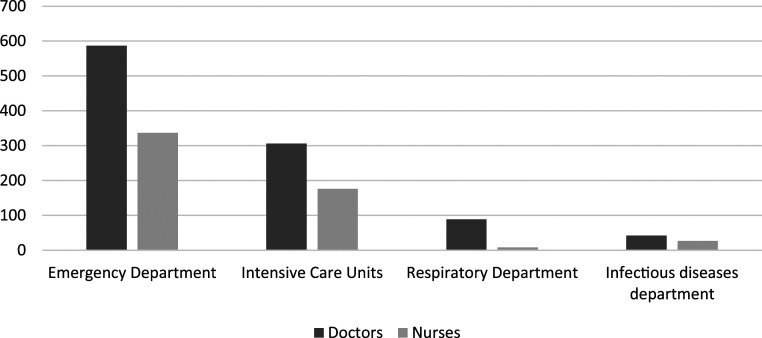
Distribution of responses by department and profession.

We obtained 1,572 valid responses out of a possible 2,000 (78.6%) participants from 21 hospitals in Libya. Among the 1,572 total healthcare personnel, 65.1% were physicians and 34.9% were nurses. Table 1 summarizes the characteristics of the study population. Figure 1 provides the distribution of participants based on departments and profession. The mean age of the population (±SD) was 35.5 ± 7.3 years for doctors and 27.8 ± 5.4 years for nurses, with women making up 64.6% of all respondents. Approximately half of the participants had low confidence regarding their ability to manage COVID-19 patients. Most participants (70% of both doctors and nurses) named social media as a source of information for COVID-19, which was statistically significant, whereas fewer than 4% viewed training courses as such.

**Table 1:** Baseline characteristics across the study population

Characteristic	Doctors (*n* = 1,024), *n* (%)	Nurses (*n* = 548), *n* (%)	χ2
Age range (years)			< 0.001†
< 35	580 (56.6)	481 (87.8)	
≥ 35	444 (43.4)	67 (12.2)	
Gender			0.156
Male	375 (36.6)	181 (33)	
Female	649 (63.4)	367 (67)	
Department			< 0.001†
Emergency department	587 (57.3)	337 (61.5)	
Intensive care units	306 (29.9)	176 (32.1)	
Respiratory department	89 (8.7)	8 (1.5)	
Infectious disease department	42 (4.1)	27 (4.9)	
Previous experience of outbreak			> 0.05
SARS	57 (5.6)	32 (5.8)	
MERS	25 (2.4)	17 (3.1)	
Bird flu	114 (11.1)	74 (13.5)	
Other	17 (1.7)	7 (1.3)	
Years of experience			0.323
< 5	477 (46.6)	241 (44)	
≥ 5	547 (53.4)	307 (56)	
Sources of information			
Media (TV and radio)	665 (65.1)	331 (60.8)	0.093
Social media	715 (70.1)	412 (75.7)	0.018*
Training courses	37 (3.6)	13 (2.4)	0.185
Discussion with colleagues	202 (19.2)	103 (17.3)	0.225
Online courses	125 (12.3)	47 (8.6)	0.03*
Confidence in management of COVID-19 patients			0.428
No confidence	510 (49.9)	275 (50.2)	
Little confidence	348 (34)	200 (36.5)	
Confidence	110 (10.8)	46 (8.4)	
High confidence	55 (5.4)	27 (4.9)	

* Significant at *P* < 0.05.

† Significant at *P* < 0.001.

Doctors had an average of 7.8 ± 7.6 years of healthcare experience, whereas nurses had an average of 5.8 ± 4.1 years. All participants worked in general public hospitals: 30.7% were in ICUs, 58.8% in the emergency department, 3.2% in the infectious disease department, and 7.4% in respiratory departments. A total of 47.3% of doctors and 54.7% of nurses did not receive adequate training in the use of PPE. Meanwhile, 16.2% of doctors and 13.3% of nurses indicated confidence in their ability to handle suspected COVID-19 patients, versus 83.8% of doctors and 86.7% of nurses who did not indicate confidence in this regard. About 45% of doctors and 37% of nurses indicated that they were not prepared to handle cases of infection transmitted at the hospital level.

The measurements of knowledge, awareness, and preparedness are presented in Table 2. The reliability of the knowledge and preparedness questionnaires was determined using Cronbach’s alpha, which revealed scores of 0.72 and 0.68 for the knowledge and preparedness scales, respectively. The results showed that less than half of all participants knew the symptoms of COVID-19 infection (44.4% of doctors and 31.7% of nurses). Similar results were obtained for case identification. However, less than 70% of the participants were aware of the mode by which COVID-19 infection was transmitted. In addition, 52.7% of doctors and 45.3% of nurses knew about PPE. Interestingly, only 43.2% of doctors and nurses were aware of proper handwashing and hand hygiene techniques to prevent the transmission of COVID-19.

**Table 2:** Percentage of correct answers on preparedness

Question type	Answered correctly, *n* (%)		
	Overall	Doctors	Nurses
Knowledge and awareness			
Q1: What are the symptoms of COVID-19 infections?	42.4	44.4	31.7
Q2: How to diagnose COVID-19	55.9	57.5	52.9
Q3: COVID-19 case definition	41	40.3	42.3
Q4: Identification of at-risk patients	59.9	61.7	56.6
Q5: How to prevent transmission of COVID-19	68.8	70.3	65.9
Q6: Knowledge of personal protective equipment (PPE)	50.1	52.7	45.3
Q7: Handwashing and type of Disinfectant	43.2	44.2	33.3
Preparedness			
Q1: Have you participated in a training course for outbreak management?	6.3	6.7	5.5
Q2: Protocol for triage and isolation of suspected cases?	25.2	23.8	38.4
Q3: Availability of isolation room?	18.2	16.1	22.1
Q4: Are you prepared to manage the COVID-19 outbreak?	22.6	20.6	26.3
Q5: Do you consider your hospital to be prepared for the COVID-19 outbreak?	13.4	13.4	13.5
Q6: Are you prepared to properly use PPE?	54.3	54	54.9
Q7: Do you know the isolation procedure?	36.1	35.6	37
Q8: Do you know how to report a potential COVID-19 case?	26.3	25	28.8
Q9: Do you know what to do if you have signs of the COVID-19 infection?	42.3	45.1	37
Q10: Do you know the safety precautions that should be taken for aerosol transmission in patients with COVID-19?	34.2	35.1	32.5
Q11: Do you know the criteria to guide the evaluation of persons under investigation?	22.1	21.2	23.9

In terms of preparedness, less than 7% of both doctors and nurses had taken courses, or training, on COVID-19. About 18% of participants reported the presence of an isolation room and the availability of a known protocol for isolation. However, only about 13% of all healthcare workers felt that hospitals were prepared for the COVID-19 outbreak. Meanwhile, 20.6% of doctors and 26.3% of nurses perceived themselves as personally prepared for COVID-19. The majority (65.8%) also reported that they were not prepared to take precautions to prevent aerosol transmission via COVID-19 patients.

The majority (73.5%) reported an inadequate level of knowledge on COVID-19. There was no significant association between hospital department type and level of participant preparedness (*P* = 0.319) or knowledge (*P* = 0.166). Tables 3 and 4 show the level of preparedness and knowledge among healthcare workers by department.

**Table 3:** Participant scores regarding preparedness (*n* = 1,572)

Department	Adequate (≥ 8), *n* (%)	Inadequate (< 8), *n* (%)	χ2
Overall	123 (7.8)	1,448 (92.2)	0.166
Emergency department	68 (7.4)	855 (92.6)	
Intensive care units	39 (8.1)	443 (91.9)	
Respiratory department	8 (6.9)	108 (93.1)	
Infectious disease department	8 (16)	42 (84)	

**Table 4:** Participant scores regarding knowledge and awareness (*n* = 1,572)

Department	High knowledge (≥ 5) *n* (%)	Low knowledge (< 5), *n* (%)	χ2
Overall	416 (26.5)	1,156 (73.5)	0.319
Emergency department	241 (26.1)	683 (73.9)	
Intensive care units	131 (27.2)	351 (72.8)	
Respiratory department	26 (22.4)	90 (77.6)	
Infectious disease department	18 (36)	32 (64)	

## DISCUSSION

A significant number of healthcare workers expressed low levels of awareness and preparedness regarding COVID-19. This raises a concern regarding the ability of the Libyan healthcare system and its healthcare workers to combat COVID-19 infection. Despite these concerns, along with the poor local healthcare infrastructure in Libya, healthcare workers continue to work during COVID-19, risking their lives to save their patients. Meanwhile, no official courses or training programs are available, and healthcare workers have to purchase PPE themselves, as they are not provided by the hospitals in adequate amounts. Our study provides considerable insights into the necessity of immediate and determined efforts focused on training programs and providing an adequate supply of PPE to alleviate these challenges during the COVID-19 pandemic.

Inadequate knowledge is a risk factor for disease transmission, as it can lead to low levels of care. Our study demonstrated that only 6.7% of doctors and 5.5% of nurses had participated in training courses. Our study also reported that about 70% of participants received information from social media, which is lower than previously reported (91.1%). Furthermore, our study indicated the lowest rate of knowledge compared with previous studies, where we found that the overall rate of respondents providing correct answers on the knowledge questionnaire was a mere 26.5%, compared with a previous study reporting that 90% of healthcare workers provided correct answers.^23^ Another recent study demonstrated that 89% of healthcare workers had sufficient knowledge on COVID-19.^24^

Another primary concern emerging from this study is that only 21.2% of doctors and 23.9% of nurses knew the criteria for evaluating persons under investigation for COVID-19 infection. In addition, only about 25% of doctors knew how to report potential COVID-19 cases, which could prompt an unexpected increase in undiagnosed cases, thereby increasing the burden of infection within the community. Moreover, approximately 18% of participants were unaware of isolation room specifications and processes for potential COVID-19 patients, which could increase the risk of infection within hospitals.

Interestingly, only 44.2% of doctors and 33.3% of nurses were aware of the proper handwashing and hand hygiene techniques and disinfectants. In addition, about 45.1% of doctors and 37% of nurses were not prepared to manage a case with signs and symptoms of COVID-19 infection. This could highlight the risk of cross-contamination within hospitals and could lead to a higher rate of hospital-acquired infections. Training and safety precautions, focused on the direct decontamination of contact points among healthcare workers, are needed to prevent the spread of infection.^25,26^

The majority of healthcare workers (77.4%) felt personally unprepared to address COVID-19 infection. A total of 50.1% of participants were uneducated about PPE, whereas about 54.3% were not trained to use it. In addition, PPE is very limited, and hospital workers reported that they independently purchase their own PPE because of the inadequate supply provided by Libyan hospitals. Furthermore, about 35.1% of doctors and 32.5% of nurses were unprepared to take safety precautions to prevent aerosol transmission via individuals with suspected COVID-19 infection. Only about 68% of the participants were aware of measures to prevent transmission of the COVID-19 virus. These issues raise fear and concerns for safety of healthcare workers and their ability to access PPE safety measures; this calls into question the healthcare system’s ability to prevent hospital-acquired infection. Hospitals can also become sites of contamination that spread COVID-19 infections into the community; furthermore, healthcare workers can also pass on the infection to family members at home.^27^

Our study found that doctors and nurses were buying PPE themselves, prompting questions around the hospitals’ inabilities to provide this essential equipment. Only a small proportion (13.4%) of participants perceived their hospital as prepared for the outbreak. More training and education are needed on the triage and isolation of suspected cases, as approximately 25% of participants are not prepared, or trained, to conduct these protocols.

### Limitations.

Although the study provided a large-scale sample from 21 healthcare centers, and used a sample size that was sufficiently large enough to allow the adequate assessment of healthcare workers’ knowledge and preparedness regarding COVID-19 infection, some limitations should still be clarified. The study was conducted in a single country with low resource levels and a lower number of detected COVID-19 infections than other countries, which may have affected the results. Future multinational studies, using more extensive and varied populations, are needed to validate these findings.

## CONCLUSION

In conclusion, our study has illuminated the current level of knowledge and awareness of COVID-19 among doctors and nurses, with special consideration for those working in departments responsible for caring for COVID-19 patients. We focused on healthcare workers who may come into direct contact with COVID-19 patients, and are thus expected to have adequate knowledge and preparedness. By contrast, other studies have focused on more general populations of healthcare workers.^23,24,28,29^ This study provides an overview of healthcare workers’ preparedness regarding the current pandemic. The respondents had a lower level of preparedness, which highlights the importance of education and training programs for healthcare workers, to control and prevent infection from COVID-19. However, the absence of an organized and effective governmental plan, along with a poor healthcare infrastructure, renders developing countries vulnerable. Moreover, educational initiatives, along with more tangible forms of support, such as the provision of PPE, should be carried out to help developing countries improve their abilities to control and prevent COVID-19 infection.
